# Psychosocial Impact and Parental Stress in Families of Children With Severe Neurological Disorders and Intellectual Disabilities: A Cross‐Sectional Study

**DOI:** 10.1002/nop2.70470

**Published:** 2026-02-23

**Authors:** Blanca Egea‐Zerolo, Ana Berástegui, Julio C. De La Torre‐Montero, Elisa Benito‐Martínez, Jorge Vidal, Noemí García‐Sanjuán, Oscar Arrogante, Ana S. F. Ribeiro

**Affiliations:** ^1^ Department of Health Sciences, San Juan de Dios School of Nursing and Physical Therapy Comillas Pontifical University Madrid Spain; ^2^ San Juan de Dios Foundation Madrid Spain; ^3^ University Institute of Family Studies Comillas Pontifical University Madrid Spain; ^4^ IPSM, Department of Child and Adolescent Psychiatry Gregorio Marañón General University Hospital Madrid Spain; ^5^ Faculty of Education and Humanities International University of La Rioja‐UNIR Madrid Spain; ^6^ Nursing Department, Faculty of Nursing, Physiotherapy, and Podiatry Complutense University of Madrid Madrid Spain

**Keywords:** conduct disorder, family, intellectual disability, neurologic disorder, psychosocial functioning

## Abstract

**Objective:**

To examine the psychosocial impact and parental stress associated with severe neurological disorders and intellectual disabilities in children, with a particular focus on the relationships between parental response styles and family stress and impact.

**Design:**

A descriptive, cross‐sectional, prospective study was conducted.

**Methods:**

The study included parents of children with neurological disorders and intellectual disabilities. Participants completed the Family Management Style Framework, Parenting Stress Index, and Parental Response Style Questionnaire. In addition, clinical characteristics related to psychological or psychiatric problems and conduct disorders were collected via parent‐reported questionnaire items and analysed as categorical variables.

**Results:**

One hundred parents participated in the survey, with data from 100 children analysed. Discomfort and Stress showed positive and significant relationships with all the factors of the Parental Response Style Questionnaire, except for Overprotection, while the Family Management Style Framework subscales did so with the Apathy/dysphoria. The presence and clinical complexity of psychological or psychiatric problems and conduct disorders were significantly associated with higher levels of parental stress and family impact.

**Conclusions:**

Families of children with intellectual disabilities face substantial challenges that affect family functioning and daily activities. The presence and clinical complexity of psychological or psychiatric problems and conduct disorders emerged as key clinical characteristics associated with increased parental stress and family impact. Given the cross‐sectional design of the study, these findings should be interpreted as associative, and no causal, directional, or temporal inferences can be drawn.

**Impact:**

Parents provided critical insights into the challenges of caring for children with severe neurological disorders and intellectual disabilities, contributing to a better understanding of family dynamics and stress‐related factors. Identifying relevant clinical characteristics may help nurses and other health professionals tailor family‐centred interventions.

**Reporting Method:**

This paper is reported according to the STROBE Statement.

**Patient or Public Contribution:**

Parents of children with severe neurological disorders and intellectual disabilities were the subjects of data collection for this study and participated in the survey after providing voluntary consent to participate.

## 1 Introduction

Intellectual disability is a dynamic concept that has evolved. According to the American Association on Intellectual and Developmental Disabilities (AAIDD), it is characterised by significant limitations in intellectual functioning and adaptive behaviour that originate before the age of 22 (Schalock et al. 2021).

Approximately 1% of the Spanish population, totalling 282,000 individuals, has been diagnosed with intellectual disability, including 52,000 children and adolescents (National Statistics Institute 2023). However, accurate epidemiological data may be challenging, as they often rely solely on an official disability certificate, thereby excluding other neurodevelopmental disorders (Ministry of Social Rights 2022).

Severe neurological disorders accompanied by intellectual disability represent complex conditions with diverse symptoms and varying degrees of severity. These conditions are often challenging to diagnose and may require extensive specialist consultations and hospitalisations (Ministry of Social Rights 2022). While extensive research exists for chronic pathologies in fields such as oncology, asthma, diabetes, and mental health, studies on families of children with neurological disorders and intellectual disabilities are limited. These studies often feature heterogeneous samples and lack differentiation by diagnosis (Svavarsdottir et al. 2014; Xu 2007).

Families with a child facing intellectual disabilities encounter challenges that significantly affect various aspects of family functioning, defined as the family's ability to fulfil roles, maintain healthy relationships, and adapt to challenges to ensure the well‐being of its members (Davis and Gavidia‐Payne 2009; Rolland 1994). This challenge is partly associated with parental stress, understood as the psychological tension resulting from the demands of raising and caring for children (Biswas et al. 2015; Yorke et al. 2018). Families with children with intellectual disabilities often experience more significant parental stress compared to those without disabilities or with other illnesses (Vela Llauradó and Riveiro 2022; Woodman et al. 2015). Parental stress interacts with the disruptions and challenges that disability imposes on daily family dynamics and routines, which have been conceptualised as family impact (Vela Llauradó and Riveiro 2022). The effect of parental stress and the impact of disability on daily life within family functioning is related to the family's coping strategies or parenting response styles. Problem‐focused coping strategies have been longitudinally associated with more significant family adjustment than emotion‐focused strategies (Benson 2010; Lai et al. 2015; Obeid 2015), while defensive strategies are linked to adverse outcomes in families dealing with various pathologies (Pitillas 2013).

The impact of disability on families varies depending on the support needs and the presence of psychological or psychiatric problems. Paradoxically, the impact is sometimes less severe with greater disability severity, although these results are not consistently replicated (Davis and Gavidia‐Payne 2009). The association of intellectual disability with mental disorders, especially conduct disorders (CD) and autism spectrum disorders (ASD), leads to a more negative impact on parents, resulting in increased feelings of fatigue, stress, depression, and lower well‐being (Baker et al. 2003; Davis and Gavidia‐Payne 2009; Efstratopoulou et al. 2022; McIntyre et al. 2002; Yorke et al. 2018).

Our study addresses an unmet need by systematically investigating the psychosocial impact and parental stress experienced by families with a child facing both neurological disorders and intellectual disabilities. Specifically, the objectives of this study were to describe levels of psychosocial impact and parental stress and to examine the relationships between these variables in families of children with neurological disorders and intellectual disabilities.

In addition, the study aimed to explore the relationships between parental response styles, family impact, and parental stress, as well as to analyse the associations between selected clinical characteristics of the child (e.g., disability‐related needs, psychological or psychiatric problems, and conduct disorders) and family functioning and parental stress.

## 2 Methods

### 2.1 Design and Participants

A descriptive, cross‐sectional, prospective study was designed involving Spanish families with children with intellectual disabilities and severe neurological disorders. The study took place between March and June 2021. The recruitment of participants for the study was carried out through the AVA Foundation. This Spanish non‐profit organisation supports people with neurological disorders and their families through comprehensive care using health and psychosocial care.

To collect the necessary data for our study, AVA Foundation distributed an informative letter to all its partners, encouraging participation in the study and providing details about its main objectives, methodology, ethical considerations, and confidentiality. Subsequently, parents who met the inclusion criteria we included those parents willing to participate in the study who had a dependent child with a neurological disorder and intellectual disability aged 30 years or less, due to their dependency on their parents received an email containing more detailed information about the objectives of the study and a link to an online questionnaire (e‐survey) created using SurveyMonkey software. In order to increase the response rate, two reminders were sent to all foundation members who had not responded: one after 2 weeks and another after 1 month from the initial email. The questionnaire was accepted by 175 subjects, of whom 64 did not complete any field (*n* = 111). Four of them had children over 30 years old, 1 did not provide information about age, and 6 of them were duplicates: finally, 100 respondents completed the questionnaire, and these responses were included and considered as valid in the final analysis. A post hoc sample size justification was conducted using G*Power version 3.1.9.6. Based on comparisons between two independent groups (presence vs. absence of psychological or psychiatric problems or conduct disorder), a two‐tailed test, an estimated large effect size (Cohen's *d* = 0.80), a significance level of *α* = 0.05, and a desired power of 0.95, a minimum sample size of 84 participants was required. Allowing for a 10% attrition rate (Arrogante 2022). The target sample size was set at 93 participants. The final sample of 100 parents exceeded this threshold.

The research team is committed to adhering to the principles outlined in the Declaration of Helsinki and data protection regulations. Before completing the questionnaire, participants were provided with an informed consent form, giving them the option to opt out if they wished. All data collected were anonymised to ensure the confidentiality of the information obtained. Furthermore, the project received approval from the Ethics and Research Committee of the Universidad Pontificia Comillas in September 2020.

### 2.2 Measures

To obtain and subsequently analyse the data needed for our study, the research team designed an ad hoc electronic questionnaire (e‐survey). This questionnaire included sociodemographic and clinical variables as well as family‐validated scales.

The assessment of support needs was conducted based on three different variables: the degree of dependency (degree 0, I, II, or III), the level of disability (mild, moderate, severe), and the number of complementary diagnoses reported by the family.

Conduct Disorder (CD) was evaluated dichotomously (no/yes) as part of a list of co‐occurring diagnoses or additional health issues alongside the primary diagnosis of disability (such as physical disability, ADHD, ASD, etc.). Psychological or psychiatric problems (PPP) were collected through parent‐reported questionnaire items and operationalised as categorical clinical characteristics, based on the type of care received. Specifically, parents indicated whether their child had received: (a) no psychological or psychiatric treatment, (b) psychotherapy only, (c) psychotherapy combined with psychotropic medication, or (d) psychotherapy combined with psychotropic medication and previous psychiatric hospitalisation. These categories were used to reflect differences in clinical complexity and treatment intensity.

Parenting Stress was evaluated using the Parenting Stress Index‐short form (PSI/SF), which assesses stress levels derived from the characteristics of the parents, the child, or the parent–child relationship (Abidin 1995). (Table 1).

**TABLE 1:** Scores achieved in the different scales: Parenting Stress Index Short‐Form (PSI/SF), Family Management Style Framework (FaMM) and CERP (Parental Response Styles Questionnaire).

Scale	Subscale (Items)	*α*	*ω*	Range	Mean	SD	*α*	*ω*
		Original scale			Our study			
*PSI/SF*	Discomfort (12)	0.90	0.87	1–5 (very Unsuitable to very suitable)	2.77	0.91	0.91	0.92
	Stress (24)	0.87	0.91		2.71	0.94	0.95	0.95
	Total	0.91	0.93		2.33	0.59	0.96	0.96
*FaMM*		Mother/Father						
	Child Daily Life (5)	0.76/0.79		1–5 (never to always)	2.45	0.95	0.75	0.76
	Condition Management Ability (12)	0.72/0.73			3.31	0.59	0.64	0.70
	Condition Management Effort (4)	0.74/0.78			3.74	0.97	0.60	0.62
	Family Life Difficulty (14)	0.90/0.91			3.14	0.94	0.91	0.91
	Parental Mutuality (8)	0.79/0.75			3.89	0.92	0.86	0.87
	View of Family Impact (10)	0.73/0.77			3.32	0.74	0.75	0.76
*CERP‐R*	Appathy/dysphoria (6)	0.795	0.801	Likert‐type scale: 1–4 (never, rarely, frequently, almost always)	2.33	0.59	0.79	0.80
	Irritability/rejection (3)	0.719	0.731		2.42	0.51	0.69	0.70
	Overprotection (4)	0.546	0.593		2.59	0.52	0.57	0.63
	Perception of maladjustment (3)	0.795	0.810		2.40	0.79	0.79	0.81

Abbreviations: ICR, internal consistency reliability; SD, standard deviation; α, Cronbach's alpha; ω, McDonalds Omega.

Family Management Style was assessed using the FaMM questionnaire (Knafl et al. 2012), which measures the impact of the child's condition on the family and the couple (Table 1).

Parental response style was evaluated using the Questionnaire of Parental Response Style in its reduced form (CERP‐R), which quantifies parental response style in families with chronically ill children through 33 items. In our study, item 7 (“When another child is having a hard time, does he try to keep your child from noticing?”) seemed to measure the inferred construct worse (without this item, the alpha value would be *α* = 0.62) (Table 1).

### 2.3 Data Analysis

#### 2.3.1 Descriptive Analysis

Initially, a descriptive analysis was developed of the different study variables. For quantitative variables, the mean, standard deviation (SD), minimum, and maximum were used, and for categorical variables, absolute and percentage frequencies were used.

#### 2.3.2 Correlation

Next, the relationship between the different variables and the scores on the subscales of the FaMM and PSI questionnaires was analysed. Given the quantitative nature of the variables studied, Pearson's correlation was used in all cases. Pearson's *r*values were interpreted as measures of the strength and direction of associations. Third, the samples were compared according to the presence of a CD or PPP. The Chi‐square test was used for categorical variables, while the Student's *t*‐test for independent samples was used for quantitative variables. The effect size was assessed according to Cohen's *d*.

#### 2.3.3 Statistical Test of Normality

The distribution of the main continuous variables was assessed using the Kolmogorov–Smirnov test, which is appropriate for samples larger than 50 participants. The results supported the assumption of approximate normality. In addition, parametric procedures such as Pearson's correlation are considered robust to moderate deviations from normality in samples of similar size (Lumley et al. 2002; Poncet et al. 2016). Therefore, Pearson correlation coefficients were deemed appropriate for the analyses conducted.

### 2.4 Software

All the data obtained was transferred to an MS Excel database and, once cleaned, to the statistical programme Jamovi (Jamovi 2021) and R software, R Core Team (2018). Finally, we worked with a confidence level of 95%, considering significant results with an associated probability value of *p* < 0.05. Given the exploratory nature of the analyses and the number of bivariate correlations performed, results should be interpreted with caution due to the potential inflation of Type I error.

## 3 Results

### 3.1 Sociodemographic Characteristics of Parents and Children

A hundred Spanish families with children under 30 years old diagnosed with intellectual disabilities and severe neurological disorders participated in the study. The respondents were 75.0% female, with a mean age of 47.0 ± 10.7 years. Seventy‐three percent of the participants had a university education, and the majority were married. Regarding income, 38.0% of the sample reported an annual family income of over €50,000, while 37.0% reported incomes between €25,000 and €50,000 (Table 2).

**TABLE 2:** Descriptive parameters of the parents included in the study (*n* = 100).

Parents' variables	*N* (%)
Level of education	
Primary education, *n* (%)	3 (3.00)
Pre‐university studies, *n* (%)	8 (8.00)
Professional Training School (FP1 or FP2), *n* (%)	16 (16.00)
University studies, *n* (%)	73 (73.00)
Family income	
< 15.000 €	13 (13.00)
Between 15,000 and 24,999 €.	9 (9.00)
Between 25,000 and 49,999 €.	37 (37.00)
> 50.000 €	38 (38.00)
Marital status	
Single	2 (2.00)
Married	73 (73.00)
Separated or divorced	12 (12.00)
Widowed	4 (4.00)
Reconstituted family	1 (1.00)
Living as a couple	8 (8.00)
Child's degree of dependency	
Currently under procedure	7 (7.00)
Grade 0 Not granted	2 (2.00)
Grade I (moderate)	11 (11.00)
Grade II (severe)	17 (17.00)
Grade III (high dependency)	44 (44.00)
Unknown	5 (5.00)
Not processed	12 (12.00)

### 3.2 Disabilities

The total number of children with disabilities in the participant families was 100, with a mean age of 14.8 ± 10.7 years, of which 66.7% were male. At the time of the survey, 17.0% of the children had a grade II degree of dependency, while 44.0% were highly dependent (grade III). Additionally, 95% of the children had a recognised degree of disability, with a mean degree of disability of 62.0%.

### 3.3 Diagnoses

The diagnoses were highly heterogeneous, with ASD (14 cases), fetal alcohol syndrome (8), tuberous sclerosis (6), Angelman syndrome (6), cerebral palsy (6), and acquired brain injury (4) being the most common in our sample. West syndrome, fragile X syndrome, Rett syndrome, Prader‐Willi syndrome, and spinocerebellar ataxia were the primary diagnoses in only one of the cases examined. In 12 cases, the aetiology was unknown, and five families did not know the diagnosis. Associated medical and mental health problems were prevalent in our sample, with language impairment (57 cases) being the most frequent. Other prevalent problems affecting our study patients included epilepsy (40), conduct disorders (34), autism spectrum disorders (34), physical disability (33), lower extremity motor limitations (29), other mental health problems (21), Attention Deficit Hyperactivity Disorder (20), visual impairment, hearing impairment/deafness, and sensory impairment (18, 6, and 16 cases, respectively), and cerebral palsy (16).

### 3.4 Relationship Between Parenting Stress Index Short‐Form (PSI/SF), family Management Style Framework (FaMM) and Parental Response Styles Questionnaire (CERP) Subscales

Significant relationships were observed between the Discomfort and Stress subscales of PSI‐SF with all CERP subscales (*p* < 0.001), except with Overprotection (Table 3). As Discomfort and Stress increased, Family Life Difficulty (*p* < 0.001), Condition Management Effort, and View of Family Impact also increased (positive correlation), while Condition Management Ability (*p* < 0.001), Child's Daily Life (*p* < 0.001), and Parental Mutuality decreased (negative correlation).

**TABLE 3:** Pearson correlation coefficients between Parenting Stress Index (PSI), Family Management Style Framework (FaMM) and Parental Response Styles Questionnaire (CERP) subscales.

Items		PSI		CERP				FaMM				
		Parental Stress	Discomfort	Apathy/dysphoria	Irritability/rejection	Over‐protection	Perception of maladjustment	Child's Daily Life	Condition Management Ability	Condition Management Effort	Family Life Difficulty	Parental Mutuality
Discomfort	*r*	0.63***	—									
Apathy/dysphoria	*r*	0.695***	0.72***	—								
Irritability/rejection	*r*	0.55***	0.36***	0.51***	—							
Overprotection	*r*	0.15	0.09	0.12	0.10	—						
Perception of maladjustment	*r*	0.75***	0.46***	0.61***	0.61***	0.17	—					
Child's Daily Life	*r*	−0.47***	−0.41***	−0.50***	−0.19	−0.15	−0.29**	—				
Condition Management Ability	*r*	−0.50***	−0.48***	−0.57***	−0.28	−0,067	−0.51***	0.52***	—			
Condition Management Effort	*r*	0.41***	0.32**	0.42***	0.15	0.03	0.40***	−0.37***	−0.37***	—		
Family Life Difficulty	*r*	0.69***	0.64***	0.63***	0.29	0.21*	0.49***	−0.73***	−0.62***	0.53***	—	
Parental Mutuality	*r*	−0.29*	−0.37**	−0.35**	−0.27*	−0.24*	−0.28*	−0.02	0.19	−0.21	−0.18	—
View of Family Impact	*r*	0.38	0.40***	0.44***	−0.06	0.08	0.23*	−0.61***	−0.55***	0.53***	0.70***	−0.08

*Note:* Values represent Pearson correlation coefficients (*r*). Statistically significant results: *p < 0.05; **p < 0.01; ***p < 0.001.

PSI‐SF scales revealed positive and significant correlations with all CERP subscales (*p* < 0.001) except for Overprotection. In contrast, FaMM subscales did so with CERP Apathy/dysphoria (Table 3) so that Apathy/dysphoria were related to greater Condition Management Effort, Family Life Difficulty and View of Family Impact and better Child's Daily Life, Condition Management Ability, and Parental Mutuality (*p* < 0.001).

### 3.5 Variables Related to Parental Stress and Family Impact

A negative relationship was observed between the degree of disability and the Parental Stress subscale (*r* = −0.25, *p* < 0.05). Conversely, higher degrees of disability, greater levels of dependence, and more diagnoses were associated with lower Child's Daily Life scores (*p* < 0.001) and higher View of Family Impact scores. Additionally, increased dependence and a higher number of diagnoses were linked to greater Condition Management Effort. These relationships were of moderate magnitude (*p* < 0.001) (Table 4).

**TABLE 4:** Pearson correlations between Parenting Stress Index (PSI) and Family Management Style Framework (FaMM) subscales and clinical characteristics of the child (*n* = 82).

		Degree of disability	Dependency level	No. of diagnoses	PPP	CD
Stress	*r*	−0.25*	−0.14	0.12	0.66***	0.52***
	*n*	74	60	82	82	82
Discomfort	*r*	−0.07	−0.07	0.15	0.35***	0.25*
	*n*	74	60	82	82	82
Child Daily Life	*r*	−0.30**	−0.40***	−0.50***	−0.40***	−0.33**
	*n*	82	67	88	88	88
Condition Management Ability	*r*	−0.06	−0.22	−0.39***	−0.31**	−0.33**
	*n*	82	67	88	88	88
Condition Management Effort	*r*	−0.03	0.27*	0.31**	0.28**	0.27*
	*n*	82	67	88	88	88
Family Life Difficulty	*r*	0.10	0.17	0.39***	0.55***	0.48***
	*n*	82	67	88	88	88
Parental Mutuality	*r*	0.21	0.20	−0.09	−0.17	−0.11
	*n*	68	55	73	73	73
View of Family Impact	*r*	0.31**	0.44***	0.46***	0.28**	0.24*
	*n*	82	67	88	88	88

*Note:* Values represent Pearson correlation coefficients (*r*). Statistically significant results: *p < 0.05; **p < 0.01; ***p < 0.001.

Likewise, the presence and clinical complexity of psychological or psychiatric problems (PPP) and the presence of conduct disorder (CD) showed positive relationships with the Discomfort and Stress subscales of the PSI‐SF (*p* < 0.001). Positive correlations were also observed with Condition Management Effort, Family Life Difficulty, and View of Family Impact, while negative associations were found with Child's Daily Life and Condition Management Ability (*p* < 0.001). These correlations were robust. In contrast, the FaMM Parental Mutuality subscale did not show significant relationships with PPP or CD (Table 4).

Overall, the variables most strongly associated with parental stress and family impact were the presence of CD and PPP, both of which reached statistical significance (*X* ^2^ (1) = 19.8; *p* < 0.001). When PPP were present, the odds of CD were 12.7 times higher: 50.0% of children with PPP also presented CD, compared with 7.0% of those without PPP.

### 3.6 Analysis of FaMM and PSI Results as a Function of the Existence of PPP or CD

Of the 95 children with disabilities on whom the data analysis was performed, the majority (58.9%) presented PPP. Of note, 76.7% of those who did have PPP were male, and a significant association was observed between the sex of the child and the presence of PPP (*X* ^2^ (1) = 4.66; *p* = 0.031). Descriptive statistics for the remaining variables are presented in Table 5. When Discomfort and Stress scores were compared according to the presence of PPP, significantly higher levels were observed in parents of children with PPP. Statistically significant differences were observed on all FaMM and PSI scales except Parental Mutuality, where the differences approached statistical significance. Effect sizes, expressed as Cohen's standardised mean difference, ranged from moderate to substantial, with particularly large effects observed for the Stress subscale (*d* = 1.26, *p* = 0.001). (Table 5).

**TABLE 5:** Results of the Parenting Stress Index (PSI) and Family Management Style Framework (FaMM) scales in children with Psychological and or psychiatric problems (PPP) and Conduct disorders (CD).

Variable (scores)	PPP				CD			
	Absent *n* = 39	Present *n* = 56	*p*	*d*‐Cohen	Absent *n* = 66	Present *n* = 31	*p*	*d*‐Cohen
Stress	3.10 (0.90)	2.20 (0.60)	< 0.001	1.26	3.40 (0.80)	2.40 (0.80)	< 0.001	1.27
Discomfort	3.10 (1.00)	2.50 (0.70)	0.002	0.70	3.20 (1.00)	2.70 (0.90)	0.022	0.55
Child's Daily Life	2.00 (0.90)	2.60 (0.90)	0.002	−0.68	1.80 (0.90)	2.50 (0.90)	0.002	−0.72
Condition Management Ability	3.20 (0.60)	3.50 (0.60)	0.014	−0.55	3.00 (0.60)	3.50 (0.60)	0.002	−0.74
Condition Management Effort	4.00 (0.90)	3.50 (1.10)	0.017	0.53	4.10 (0.80)	3.60 (1.00)	0.012	0.58
Family Life Difficulty	3.50 (0.90)	2.70 (0.80)	< 0.001	0.92	3.80 (0.80)	2.80 (0.90)	< 0.001	1.14
Parental Mutuality	3.80 (1.00)	4.20 (0.70)	0.070	−0.44	3.90 (0.80)	4.10 (0.90)	0.378	−0.22
View of Family Impact	3.50 (0.70)	3.10 (0.80)	0.025	0.50	3.60 (0.80)	3.20 (0.70)	0.027	0.51

*Note:* Data are represented as mean (SD). Effect sizes are reported as Cohen's *d*.

The parents who participated in the survey were asked whether their child had a CD besides the disability. Three parents did not respond to this question, so of the 97 children with disabilities, the majority (68.00%) stated that their child did not have a CD. In this case, no association was observed between the child's sex and the existence of CD (*X* ^2^ (1) = 0.79; *p* = 0.373).

When comparing Discomfort and Stress data between children with and without CD, a higher degree of Discomfort and Stress was found in parents of children with CD (Cohen's *d* = 1.27, *p* = 0.001; Cohen's *d* = 1.27, *p* = 0.001 respective). Statistically significant differences were noted for all FaMM scales except for Parental Mutuality. Effect sizes, presented under Cohen's standardised mean difference, ranged from moderate to large. Particularly noteworthy were the differences in stress and FaMM Family Life Difficulty scale (Table 5).

## 4 Discussion

Most studies in families of children with chronic pathologies are conducted in oncological, asthmatic, or diabetic patients, with research in families of children with severe neurological disorders and intellectual disability being infrequent. Therefore, our study provides a broad national sample of families with a severe level of disability, a significant degree of dependence, and a high complexity of diagnoses (more than 50.0% had ≥ 5 diagnoses or associated health problems). The heterogeneity of conditions, including several rare diseases, further reflects the clinical complexity of this population.

Our study showed that families with children with severe neurological damage and intellectual disability face several specific challenges that are associated with important difficulties in family system functioning and activities of daily living in line with the preceding literature (Obeid 2015; Yorke et al. 2018). Levels of parental stress in our sample were higher than those reported in normative (Abidin 1995) and higher than those observed in Spanish families of children with Down syndrome, although lower than those described in some samples of families of children with autism (Pozo Cabanillas et al. 2006; Rubio Guzmán et al. 2022).

Regarding disability, our findings confirmed the paradoxical effect where more severe disability correlated with lower family stress (Davis and Gavidia‐Payne 2009). However, these results were not replicated concerning the degree of dependence or the number of diagnoses, nor with other variables of stress or family impact suggesting that objective disability characteristics alone do not fully explain family stress or adaptation processes (Jenaro et al. 2020). Data analysis revealed relationships between a more defensive family response style and a more negative impact on the family, consistent with studies on other pathologies (Pitillas 2013). The Apathy/Dysphoria dimension shows a distinct pattern of correlations, aligning negatively with positive dimensions of impact (Child Daily Life, Management Ability, and Parental Mutuality) and positively with negative dimensions (Effort, Difficulty, and Impact). This suggests that emotional disengagement and low motivation may be linked to poorer parental ability to manage caregiving tasks and maintain positive relational dynamics within the family. At the same time, these states are associated with more negative perceptions of caregiving as burdensome and challenging, highlighting their detrimental role in family functioning. It is worth highlighting the lack of an expected association between overprotection and family impact. This result suggests that overprotection may operate as a distinct parental behaviour, less influenced by general stress levels and more related to a specific caregiving strategy aimed at controlling or shielding the child. Unlike emotionally driven response styles, overprotection may function as a behavioural adaptation rather than a manifestation of psychological distress, highlighting the complex balance between protection and autonomy in contexts of high support needs (Callus et al. 2019). The relationship of family impact with support needs was weaker than with the presence and clinical complexity of psychological or psychiatric problems (PPP) and the presence of conduct disorder (CD). These conditions were strongly associated with higher parental stress and a more negative family impact, consistent with previous findings (Périsse et al. 2010). In our sample, nearly half of the children with neurological disorders also presented PPP, with a significantly higher prevalence among boys than girls, in line with existing literature (Périsse et al. 2010).

In interpreting these findings, it is important to note that our analyses were exploratory and based on Pearson correlations, aimed at describing the pattern and magnitude of associations rather than testing predefined hypotheses. Although we did not apply conservative corrections such as Bonferroni, the moderate‐to‐large correlation coefficients observed reduce the likelihood that the significant associations reflect false positives. Nonetheless, these results should be considered preliminary, and future confirmatory studies with larger samples and appropriate multiple‐comparison corrections are warranted to further validate and refine these associations.

Taken together, these findings suggest that one of the most substantial burdens faced by these families is related to the presence of PPP and CD. Hence, strategies should be delivered and developed to facilitate access to evaluations and treatment, reduce the stigma associated with psychiatric and psychological care, and support families in dealing with the grief sometimes involved in visiting a psychiatric clinic. In addition, it would be advisable to work with these families on feelings of failure and or guilt (Whittle et al. 2019) and encourage them to receive support for their mental health needs. Furthermore, focusing on the family's resilience may be particularly beneficial, as evidence indicates that resilience is associated with lower levels of anxiety, stress, and depression among parents of children with autism (Flores‐Buils et al. 2023).

Although individuals with intellectual disabilities may present with the same mental disorders as those without disabilities, there are more challenges with their recognition, diagnosis, and treatment. This is because their presentations differ from standard ones, necessitating greater specialisation of mental health professionals in this field (Whittle et al. 2018).

At the time of data collection, most participating parents were married and had a university education, with a substantial proportion reporting high household incomes. This profile suggests a potential association between membership in support organisations and access to information and resources, likely influenced by socioeconomic factors (Pitillas et al. 2024).

Regarding the limitations of the study, there was a sociodemographic bias in sample selection since all parents who responded to the survey were members of the Foundation, limiting the ability to extrapolate the results to the general population. From the cultural point of view, membership in associations is related to a certain tendency toward collaboration and special access to support resources. Additionally, data collection was conducted online, which may have limited the participation of some families, particularly those with lower digital literacy. Future studies could benefit from using alternative or mixed data collection methods. Furthermore, the sample size did not allow the application of more complex multivariable models to estimate the unique contribution of each psychosocial dimension. As a result, the findings should be interpreted at the level of bivariate associations, and caution is required when considering the strength or direction of these relationships. Larger samples in future studies would allow more robust multivariable modelling and improve the interpretability of these associations.

Finally, this study lacked a comparison with normative families, making it difficult to determine whether family dynamics in coping with parenting or behavioural problems differ from those in normative families.

### 4.1 Implications for Healthcare Practice and Education

In the context of paediatric care, the findings of this study reinforce the need to move from a predominantly patient‐centred approach toward a family‐centred care model (Kim and Turnbull 2004). This shift is particularly relevant for children with severe neurological disorders and intellectual disabilities, as the presence of psychological or psychiatric problems (PPP) and conduct disorders (CD) was strongly associated with increased parental stress and greater family impact, as reflected in the Family Management Measure (FaMM) results. Notably, a substantial proportion of the sample presented co‐occurring PPP and CD, highlighting the cumulative burden experienced by families.

From a healthcare perspective, these findings support the consideration of the family or caregiving system as the primary unit of care, rather than focusing exclusively on the individual with a disability. Nursing and multidisciplinary interventions aimed at fostering “sustainable family caregiving” should therefore be prioritised (Canga 2013). Such interventions may include systematic assessment of family needs, psychological support for caregivers, and coordinated care strategies that address both the child's condition and the family's overall functioning.

In addition, this study underscores the importance of incorporating theoretical frameworks that position the family as the central unit of analysis and care within healthcare organisations and educational institutions. Integrating family‐centred care principles into undergraduate and postgraduate nursing curricula can enhance professionals' competencies in disability care, promote holistic clinical decision‐making, and improve the quality of care provided to children with complex needs and their families.

## 5 Conclusions

This study shows that families of children with chronic neurological conditions and intellectual disabilities face multiple challenges that substantially affect family functioning and activities of daily living. The findings highlight the presence of psychological or psychiatric problems and conduct disorders as key clinical characteristics associated with higher levels of parental stress and greater family impact.

Although individuals with intellectual disabilities may experience mental health conditions similar to those observed in the general population, their identification and management often require a multidisciplinary approach due to atypical symptom presentation and complex care needs. This added complexity underscores the importance of specialised training for mental health and nursing professionals, as well as adequate resource allocation to support families effectively. Interventions such as targeted mental health support, family counselling, and resources to address psychosocial challenges may help mitigate the burden experienced by caregivers.

The study also highlights the need for future research to adopt a broader family perspective, incorporating the experiences of all family members, including siblings. Such an approach would allow for a more comprehensive understanding of how psychological and psychiatric problems relate to family dynamics over time. From a policy and practice perspective, the implementation of targeted training programs in mental health and paediatric care may contribute to improving the identification of needs and the delivery of appropriate support for these families.

In summary, this study sheds light on the complex realities faced by families of children with severe neurological disorders and intellectual disabilities and emphasises the necessity of family‐centred, multidisciplinary interventions tailored to their specific needs. Given the cross‐sectional nature of this study, the observed relationships should be interpreted as associations, and no conclusions about causality, directionality, or temporality can be drawn.

## Author Contributions

Conceptualisation: Blanca Egea‐Zerolo; Julio C. De La Torre‐Montero; Ana Berástegui; Ana S.F. Ribeiro. Methodology: Blanca Egea‐Zerolo; Julio C. De La Torre‐Montero; Ana Berástegui; Ana S.F. Ribeiro; Oscar Arrogante. Data curation: Blanca Egea‐Zerolo; Julio C. De La Torre‐Montero; Elisa Benito‐Martínez; Jorge Vidal; Noemí García‐Sanjuán. Formal analysis: Oscar Arrogante; Ana S.F. Ribeiro. Investigation: Blanca Egea‐Zerolo; Julio C. De La Torre‐Montero; Elisa Benito‐Martínez; Jorge Vidal; Noemí García‐Sanjuán; Ana Berástegui. Writing – original draft: Blanca Egea‐Zerolo; Ana S.F. Ribeiro. Writing – review and editing: All authors. Supervision: Ana S.F. Ribeiro. Project administration: Blanca Egea‐Zerolo. All authors approved the final version of the manuscript and agree to be accountable for all aspects of the work.

## Funding

The authors have nothing to report.

## Conflicts of Interest

The authors declare no conflicts of interest.

## Data Availability

The data supporting this study's findings are available from the corresponding author upon a reasonable request.
